# Next generation DNA sequencing technology delivers valuable genetic markers for the genomic orphan legume species, *Bituminaria bituminosa*

**DOI:** 10.1186/1471-2156-12-104

**Published:** 2011-12-15

**Authors:** María Pazos-Navarro, Mercedes Dabauza, Enrique Correal, Kelly Hanson, Natasha Teakle, Daniel Real, Matthew N Nelson

**Affiliations:** 1 Departamento de Biotecnología y Protección de cultivos, Instituto Murciano de Investigación y Desarrollo Agrario y Alimentario (IMIDA), C/Mayor s/n, 30150-La Alberca, Murcia, Spain; 2 Departamento de Recursos Naturales, IMIDA. C/Mayor s/n, 30150-La Alberca, Murcia, Spain; 3 Australian Genome Research Facility, Level 5 Gehrmann Laboratories, Research Road, University of Queensland, St Lucia, QLD 4072, Australia; 4 Centre for Ecohydrology, The University of Western Australia, 35 Stirling Highway, Crawley, WA 6009, Australia; 5 School of Plant Biology, Faculty of Natural and Agricultural Sciences, The University of Western Australia, 35 Stirling Highway, Crawley, WA 6009, Australia; 6 Department of Agriculture and Food, Western Australia, South Perth, WA 6151, Australia; 7 Future Farm Industries Cooperative Research Centre, The University of Western Australia, 35 Stirling Highway, Crawley, WA 6009, Australia

## Abstract

**Background:**

*Bituminaria bituminosa *is a perennial legume species from the Canary Islands and Mediterranean region that has potential as a drought-tolerant pasture species and as a source of pharmaceutical compounds. Three botanical varieties have previously been identified in this species: *albomarginata, bituminosa *and *crassiuscula*. *B. bituminosa *can be considered a genomic 'orphan' species with very few genomic resources available. New DNA sequencing technologies provide an opportunity to develop high quality molecular markers for such orphan species.

**Results:**

432,306 mRNA molecules were sampled from a leaf transcriptome of a single *B. bituminosa *plant using Roche 454 pyrosequencing, resulting in an average read length of 345 bp (149.1 Mbp in total). Sequences were assembled into 3,838 isotigs/contigs representing putatively unique gene transcripts. Gene ontology descriptors were identified for 3,419 sequences. Raw sequence reads containing simple sequence repeat (SSR) motifs were identified, and 240 primer pairs flanking these motifs were designed. Of 87 primer pairs developed this way, 75 (86.2%) successfully amplified primarily single fragments by PCR. Fragment analysis using 20 primer pairs in 79 accessions of *B. bituminosa *detected 130 alleles at 21 SSR loci. Genetic diversity analyses confirmed that variation at these SSR loci accurately reflected known taxonomic relationships in original collections of *B. bituminosa *and provided additional evidence that a division of the botanical variety *bituminosa *into two according to geographical origin (Mediterranean region and Canary Islands) may be appropriate. Evidence of cross-pollination was also found between botanical varieties within a *B. bituminosa *breeding programme.

**Conclusions:**

*B. bituminosa *can no longer be considered a genomic orphan species, having now a large (albeit incomplete) repertoire of expressed gene sequences that can serve as a resource for future genetic studies. This experimental approach was effective in developing codominant and polymorphic SSR markers for application in diverse genetic studies. These markers have already given new insight into genetic variation in *B. bituminosa*, providing evidence that a division of the botanical variety *bituminosa *may be appropriate. This approach is commended to those seeking to develop useful markers for genomic orphan species.

## Background

*Bituminaria bituminosa *(L.) C.H. Stirt., commonly known as Tedera in the Canary Islands, is a perennial legume species widely distributed in the Mediterranean Basin and Macaronesia. It is a self-pollinated diploid species (2n = 20) with DNA content estimated to be between 0.998 and 1.094 pg DNA per diploid nucleus [1-3]. *B. bituminosa *shows particularly high diversity in the Canary Islands, with three recognised botanical varieties: (i) var. *albomarginata*: native to semi-arid habitats in coastal areas of Lanzarote island and a few other niches in Fuerteventura, Tenerife and Gran Canaria, with an annual rainfall of 150 mm to 300 mm; (ii) var. *crassiuscula*: native to high altitude sub-humid areas in Tenerife island with up to 500 mm rainfall per year; and (iii) var. *bituminosa *widely distributed in all islands across varying altitudes and rainfall levels [4,5]. In the Mediterranean basin, only var. *bituminosa *is found [6]. These botanical varieties were identified using morphological characteristics and were largely supported by preliminary molecular analyses using arbitrary DNA markers [7,8]. However, Juan et al. [7] found that accessions of var. *bituminosa *from the Mediterranean region formed a cluster distinct from a Canary Islands cluster that contained all three botanic varieties. Therefore, there is some ambiguity in botanical variety definitions that warrants further investigation.

In recent years, there has been growing international interest in *B. bituminosa *as a potential source of pharmaceutical compounds and also as a drought tolerant pasture species. The plant contains secondary compounds such as pterocarpans with antitumor activity against leukaemia and colon cancer [9-11], antioxidants [12] and furanocoumarins such as psoralen and angelicin, which are used in the treatment of skin diseases (psoriasis, vitiligo, melanoma) [13-15]. As a forage crop it is well adapted to high temperature and low rainfall. An important attribute of this species is that, unlike lucerne (*Medicago sativa *L.), it has a high retention of leaves when moisture stressed, therefore providing valuable feed over summer [16,17]. Traditionally, the profitability and sustainability of livestock industries in southern Australia and in other regions with Mediterranean-like climates is severely constrained by the quantity and quality of forage available over summer and autumn. Therefore, there is strong demand for breeding drought-tolerant and productive forage legumes as well as improved understanding of the genetic basis of key agronomic traits.

Molecular markers contribute valuable support to breeding programmes [18]. Markers provide the means to characterise genetic diversity within breeding programmes and help identify new genetic diversity in the wild or in germplasm collections. Markers are valuable in determining or confirming pedigrees and for marker-assisted selection of traits that are difficult and/or expensive to measure. However, *B. bituminosa *can be considered a true 'genomic orphan' [19] in that there are almost no genomic resources or high-quality codominant markers available for genetic analysis. The few genomic resources readily available for molecular marker development for *B. bituminosa *consist largely of chloroplast gene sequences developed for phylogenetic studies within the tribe Psoraleeae and more broadly among phaseoloid legumes [20,21]. However, *B. bituminosa *has a rich cousin in the genomic resources sense: soybean (*Glycine max *(L.) Merr.) that belongs to the neighbouring subtribe *Glycininae *[21]. The complete genome sequence of soybean was recently determined [22], which could act as a useful reference genome for *B. bituminosa*. However, the taxonomic divide between these species is sufficiently wide to make marker transfer between soybean and *B. bituminosa *rather inefficient. Fortunately, the advent of new high-throughput genome sequencing technologies provides a relatively low cost opportunity for rapid development of locus-specific markers for a species like *B. bituminosa *that has little available genomic resources.

This study reports the generation of a cDNA library developed from leaf mRNA from a single *B. bituminosa *plant and sampling of the leaf transcriptome using 454 GS-FLX pyrosequencing technology. Simple sequence repeat (SSR) motifs were identified, primers designed and a subset of these markers were used to characterise a broad set of *B. bituminosa *accessions to assist in the correct choice of parents in breeding programmes, and which could be used to provide guidance in managing and conserving germplasm collections. These SSR markers along with the first catalogue of expressed genes provide valuable resources for *B. bituminosa *genetic analysis and breeding.

## Results

### Sampling the *B. bituminosa *leaf transcriptome by Roche 454 sequencing

Sequencing of the *B. bituminosa *leaf-derived cDNA library on the GS-FLX System resulted in 432,306 sequence reads with an average length of 345 bp (149.1 Mbp). These sequence reads were deposited at the Sequence Read Archive (SRA) database at NCBI [GenBank:SRA037309]. GS De Novo Assembler software assembled 266,461 (61.6%) of the reads into 4,542 contigs that were ≥ 100 bp in length. It then grouped contigs into 2,929 "isogroups" (analogous to genes) and 3,798 "isotigs" (analogous to transcripts) with an average isotig length of 707 base pairs. 3657 isotigs and remaining singleton contig sequences that were ≥ 200 bp in length were deposited at NCBI Transcriptome Shotgun Assembly (TSA) database [GenBank:JL856153-JL859809].

### Functional characterisation of expressed gene sequences

**Figure 1:**
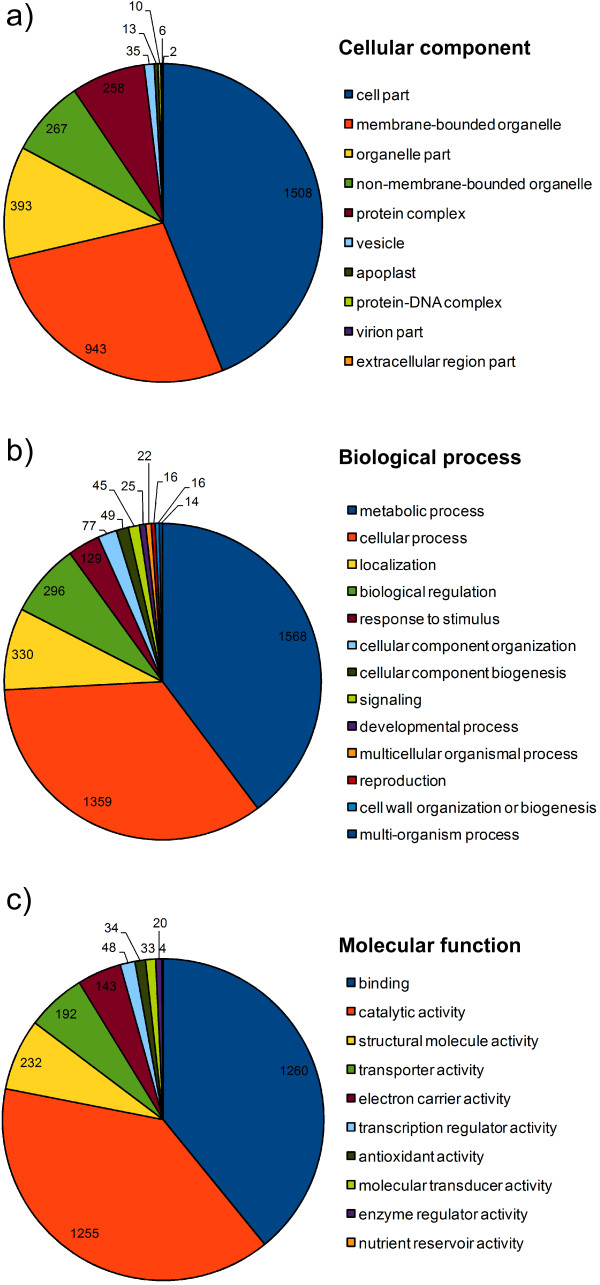
**Gene ontology (GO) terms for 3,419 expressed gene sequences obtained by Roche 454 sequencing of leaf mRNA of *Bituminaria bituminosa *plant 13.1**. GO descriptors are categorised according to: a) cellular components (level 3 terms); b) biological process (level 2 terms with > 10 sequences); and c) molecular function (level 2 terms).

3,838 isotigs and remaining singleton contig sequences ≥ 100 bp were subjected to *in silico *functional characterisation using Blast2GO software. Gene ontology (GO) terms were identified for 3,419 sequences (89.1%; Additional file 1). Figure 1 provides a summary of the main GO terms defined according to the cellular component, biological process and molecular function associated with these 3,419 sequences. The species that received the greatest proportion of BLAST hits was *Glycine max *(3,013 hits, or 88.1%), and *G. max *was also the species that was most frequently the top hit (for 1,454 sequences; 42.5%). The other main model legume species only rarely provided the most significant matches: *Medicago truncatula *was the top hit for 185 sequences (5.4%) and *Lotus japonicus *was the top hit for 14 sequences (0.4%). Together, these results indicate that *G. max *will serve as the most representative reference genome for *B. bituminosa*.

### SSR primer design and testing

With the aid of QDD software, primer pairs for 186 'perfect' SSRs and 54 'compound' SSRs were designed (Additional file 2). Of these, 49 primer pairs for perfect SSRs and 38 primers pairs for compound SSRs were selected in descending order of SSR motif length along with a further 7 primer pairs were designed manually with the aid of Geneious software using contigs containing SSR motifs (Additional file 3). In total, 94 primer pairs were synthesised and screened for amplification efficiency using genomic DNA from *B. bituminosa *plant A13.1 as template. Without any specific optimisation, 82 out of 94 primer pairs (87.2%) amplified one or more bands (visualised on low-resolution 1% agarose gels), with one band being more common (78 primer pairs, 83.0%) than ≥ 2 bands (4 primer pairs, 4.3%) (Additional file 3).

The 82 primer pairs that gave amplification products were screened for gross-level polymorphism in eight diverse *B. bituminosa *accessions (one plant per accession) by electrophoresis using 2% agarose gels. Of the 82 primer pairs tested in this way, 21 showed clear band size polymorphism and were selected for synthesis of fluorescently labelled forward primers (Table 1). These 21 fluorescently labelled primer pairs were used to genotype 79 *B. bituminosa *accessions (one plant per accession). These accessions were classed as 'original populations' (collected from wild or from traditional pasture lands; n = 27) or 'breeding lines' (having undergone selection and possible uncontrolled cross-pollination within the Spanish breeding programme; n = 52) (Table 2).

**Table 1:** *Bituminaria bituminosa *simple sequence repeat (*Bbit*-SSR) primer pairs selected for characterisation of *B. bituminosa *germplasm

Marker name	Forward primer sequence	Reverse primer sequence
*Bbit*-SSR004	ACCACCCGCAGTTACTTTCACCT	CCTTGTGCTGGTTTCACGCAACG

*Bbit*-SSR005	ACCAAGTCAGGCTGGAACCCCA	GTCCTGGCCCACTGAACGCC

*Bbit*-SSR008	CATTGACATCCCTAAGCATAATGT	TCGTTAATAGCGGTCTTGGG

*Bbit*-SSR010	GCAGGCTTTCCTGAACTGAC	GTCTCCACCAGCAATACCGT

*Bbit*-SSR012	TCATCCCTTCTCTTCCTACTCG	CGGTTTCTTCGAATACACAGTA

*Bbit*-SSR013	GAAGGCAAGTGAAAAGCCAG	TCAGACACCAGTGGCTCAAC

*Bbit*-SSR015	GACTGCACGGTCTTCTCGAC	ATGTGCAGAGGCATTTGTTG

*Bbit*-SSR034	CAATCCCATTTTCCGCTTTA	TGCCCTCTTCCTTCATAGGTT

*Bbit*-SSR035	ATATCCACCACCTTCCGTGA	GTAGGATAGGGTCCGGTGGT

*Bbit*-SSR040	TAACCACTTGGAACTGGGGT	AATTGCAACAGCAGCAACAG

*Bbit*-SSR055	AGCATCACTACGACCATCCC	GGTGACAACAGAGTGGTCTGA

*Bbit*-SSR056	TCATCCCTTCTCTTCCTACTCG	CGGTTTCTTCGAATACACAGTA

*Bbit*-SSR059	CATTGACATCCCTAAGCATAATGT	TCGTTAATAGCGGTCTTGGG

*Bbit*-SSR064	TTGCTTCTGCGTAACTGTGG	AAAAGTCCACGTCAGCATCC

*Bbit*-SSR066	GGTCGTCCCATTTATCGAAG	GGAAGAACGGTCAATGGAGA

*Bbit*-SSR067	TCACCTTCCTCACAAACTACCA	TGAAATGCCTCAATGAGCTAAA

*Bbit*-SSR070	TGTCGAACTGTTGGATTGTGA	AATTGCAACAGCAGCAACAG

*Bbit*-SSR073	TTTGCTTGTGTCCTGTCCAA	CCTTCCCTTACCCACCAAGT

*Bbit*-SSR076	AGAAGGCAAGTGAAAAGCCA	TCAGACACCAGTGGCTCAAC

*Bbit*-SSR079	GAGCTTCGGAGGGAGTTCTT	CCAAAATCCATCACCTTCCA

*Bbit*-SSR090	CCCTAACATTGGTAACAGCCA	GAGGCTGGCATCAAGTCAAC

**Table 2:** Single *Bituminaria bituminosa *plants sampled from 79 accessions from original populations (n = 27) or breeding lines (n = 52)

Accession code^1^	Botanical variety	Geographical origin	Population type^2^	Heterozygosity^3^
A1.1	*albomarginata*	Canary Islands (Famara, Lanzarote)	Breeding line	24%

A2.1	*albomarginata*	Canary Islands	Breeding line	24%

A3.2	*albomarginata*	Canary Islands	Breeding line	25%

A4.2	*albomarginata*	Canary Islands	Breeding line	5%

A5.1	*albomarginata*	Canary Islands (Famara, Lanzarote)	Breeding line	10%

A6.2	*bituminosa*	Canary Islands (Teno, Tenerife)	Breeding line	33%

A7.2	*bituminosa*	Canary Islands (Teno, Tenerife)	Breeding line	24%

A8.1	*albomarginata*	Canary Islands	Breeding line	15%

A9.1	*albomarginata*	Canary Islands (Famara, Lanzarote)	Breeding line	5%

A10.2	*albomarginata*	Canary Islands	Breeding line	0%

A11.17	*albomarginata*	Canary Islands (Famara, Lanzarote)	Breeding line	14%

A12.3	*bituminosa*	Canary Islands (Teno, Tenerife)	Original population	0%

A13.1	*bituminosa*	Canary Islands (Teno, Tenerife)	Breeding line	29%

A14.1	*albomarginata*	Canary Islands (Malpaso, Lanzarote)	Original population	19%

A15.4	*albomarginata*	Canary Islands (Famara, Lanzarote)	Original population	10%

A16.2	*crassiuscula*	Canary Islands (Vilaflor, Tenerife)	Breeding line	10%

A17.2	*crassiuscula*	Canary Islands (Cañadas del Teide, Tenerife)	Breeding line	12%

A18.8	*albomarginata*	Canary Islands (Vinamar, Fuerteventura)	Original population	0%

A19.3	*crassiuscula*	Canary Islands (Tenerife)	Breeding line	24%

A20.1	*albomarginata*	Canary Islands (Famara, Lanzarote)	Breeding line	19%

A21.1	*albomarginata*	Canary Islands (Famara, Lanzarote)	Breeding line	38%

A22.2	*albomarginata*	Canary Islands (Famara, Lanzarote)	Original population	0%

A23.2	*bituminosa*	Canary Islands (Teno, Tenerife)	Original population	28%

A24.3	*bituminosa*	Canary Islands (Teno, Tenerife)	Original population	0%

A26.1	*bituminosa*	Canary Islands (Tenerife)	Original population	25%

A27.2	*crassiuscula*	Canary Islands (Teide, Tenerife)	Original population	33%

A29.2	*albomarginata*	Canary Islands (Famara, Lanzarote)	Original population	6%

A36.1	*albomarginata*	Canary Islands (Famara, Lanzarote)	Original population	27%

A37.2	*albomarginata*	Canary Islands (Malpaso, Lanzarote)	Original population	27%

A38.3	*bituminosa*	Canary Islands (Tefia, Fuerteventura)	Original population	14%

A39.2	*albomarginata*	Canary Islands (Vinamar, Fuerteventura)	Original population	0%

A40.1	*bituminosa*	Canary Islands (Bentacuria, Fuerteventura)	Original population	21%

A41.2	*bituminosa*	Canary Islands (Güimar, Tenerife)	Original population	5%

A43.2	*bituminosa*	Canary Islands (Tenerife)	Breeding line	38%

A44.1	*albomarginata*	Canary Islands	Breeding line	10%

A46.1	*albomarginata*	Canary Islands	Breeding line	10%

A48.2	*albomarginata*	Canary Islands (Famara, Lanzarote)	Breeding line	52%

A49.3	*crassiuscula*	Canary Islands	Breeding line	52%

A50.1	*albomarginata*	Canary Islands (Famara, Lanzarote)	Breeding line	15%

A51.2	*crassiuscula*	Canary Islands	Breeding line	19%

A52.1	*albomarginata*	Canary Islands	Breeding line	20%

A53.2	*albomarginata*	Canary Islands	Breeding line	38%

A54.1	*albomarginata*	Canary Islands	Breeding line	29%

A55.2	*albomarginata*	Canary Islands	Breeding line	35%

A56.2	*bituminosa*	Canary Islands (Teno, Tenerife)	Breeding line	37%

A58.2	*albomarginata*	Canary Islands (Famara, Lanzarote)	Breeding line	52%

A62.1	*albomarginata*	Canary Islands	Breeding line	43%

A63.1	*crassiuscula*	Canary Islands (Vilaflor, Tenerife)	Breeding line	10%

A64.2	*bituminosa*	Canary Islands	Breeding line	43%

A65.1	*bituminosa*	Mediterranean region (Calnegre, Murcia, Spain)	Breeding line	0%

S1b	*bituminosa*	Mediterranean region (Llano del Beal, Murcia, Spain)	Breeding line	95%

S3c	*bituminosa*	Canary Islands (Tahonilla, Tenerife)	Original population	29%

S4a	*bituminosa*	Canary Islands (Tahonilla, Tenerife)	Original population	42%

S6c	*bituminosa*	Mediterranean region (La Perdiz, Murcia, Spain)	Breeding line	48%

S8a	*albomarginata*	Canary Islands (Famara, Lanzarote)	Original population	62%

S9a	*bituminosa*	Mediterranean region (Calnegre, Murcia, Spain)	Breeding line	68%

S10b	*bituminosa*	Mediterranean region (Sardinia, Italy)	Original population	24%

S11b	*bituminosa*	Mediterranean region (La Unión, Murcia, Spain)	Breeding line	62%

S13c	*bituminosa*	Mediterranean region (Calnegre, Murcia, Spain)	Breeding line	30%

S14b	*bituminosa*	Mediterranean region (Israel)	Original population	33%

S17b	*bituminosa*	Mediterranean region (La Unión, Murcia, Spain)	Original population	0%

S18b	*bituminosa*	Mediterranean region (La Unión, Murcia, Spain)	Original population	5%

S19b	*bituminosa*	Mediterranean region (La Unión, Murcia, Spain)	Original population	5%

S20b	*bituminosa*	Mediterranean region (La Perdiz, Murcia, Spain)	Breeding line	48%

S21c	*albomarginata*	Canary Islands (Famara, Lanzarote)	Breeding line	67%

S23b	*bituminosa*	Mediterranean region (La Perdiz, Murcia, Spain)	Breeding line	95%

S29b	*bituminosa*	Canary Islands (Tenerife)	Breeding line	15%

S30b	*bituminosa*	Canary Islands (Tenerife)	Breeding line	0%

S31b	*bituminosa*	Mediterranean region (Spain)	Breeding line	50%

S32c	*bituminosa*	Canary Islands (Tenerife)	Breeding line	29%

S33b	*albomarginata*	Canary Islands	Breeding line	24%

S34c	*bituminosa*	Mediterranean region (La Perdiz, Murcia, Spain)	Breeding line	38%

S35c	*bituminosa*	Mediterranean region (La Perdiz, Murcia, Spain)	Breeding line	63%

S36a	*bituminosa*	Mediterranean region (La Perdiz, Murcia, Spain)	Breeding line	35%

S37c	*bituminosa*	Mediterranean region (La Perdiz, Murcia, Spain)	Breeding line	55%

S38d	*bituminosa*	Mediterranean region (Greece)	Original population	11%

S39c	*bituminosa*	Mediterranean region (Greece)	Original population	5%

S40d	*bituminosa*	Mediterranean region (Greece)	Original population	11%

S41c	*bituminosa*	Canary Islands (La Palma)	Breeding line	28%

^1^Accession names prefixed by 'A' were provided by the Australian breeding programme, while accession names prefixed by 'S' were provided by the Spanish breeding programme.

^2^The term 'breeding line' indicates that the accession has been grown for one or more generation in breeding nurseries with possible cross-pollination among accessions. The term 'original population' indicates that seed was collected directly from geographical location indicated with no opportunity for cross-pollination among these accessions.

^3^Heterozygosity was assessed at 21 simple sequence repeat loci.

Fragment analysis using GeneMarker software revealed that 20 primer pairs gave clear peaks; the remaining primer pair gave variable amplification strength and was consequently omitted from subsequent analysis. Of the 20 high-quality SSR primer pairs, 19 appeared to detect single loci (1 to 2 alleles per primer pair) while one primer pair appeared to detect two loci (2 to 4 alleles for primer pair '*Bbit*-SSR079'). In total, 130 alleles were detected at 21 high-quality marker loci, an average of 6.19 alleles per locus indicating that these markers were generally highly polymorphic. The 19 single locus SSR markers detected between 3 to 11 alleles per marker, with polymorphic index content (PIC) values ranging from 0.13 to 0.76 (average = 0.407) (Additional file 4).

### Validating SSR markers by genetic diversity analysis in 27 original populations

**Figure 2:**
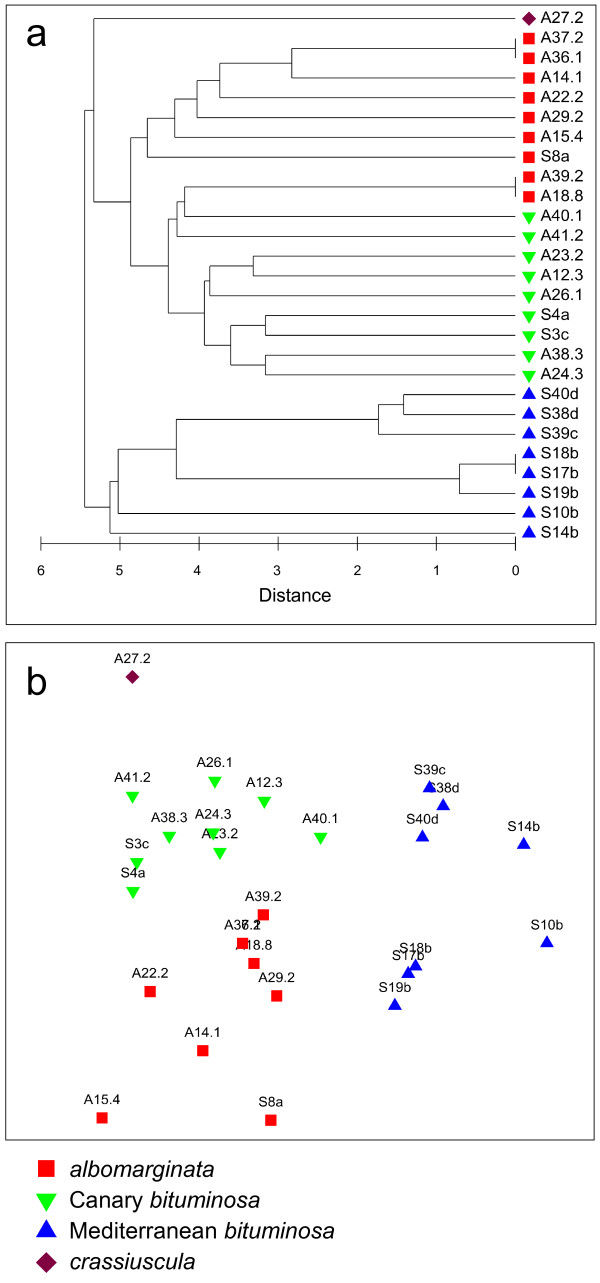
**Genetic relationships among 27 *Bituminaria bituminosa *accessions from three botanical varieties collected from the Canary Islands and the Mediterranean region**. One plant from each accession was assessed using 21 simple sequence repeat loci with the resulting 130 alleles used to calculate Euclidean pairwise distances. These distances are presented in: a) Dendrogram produced by hierarchical clustering analysis; b) Multi-dimensional scaling (MDS) plot (2D stress = 0.16).

To determine if the SSR markers were suitable for inferring genetic relationships among breeding lines, a pairwise Euclidean distance matrix for 27 accessions collected from the wild or from traditional pasture-lands (termed 'original populations'; Table 2) was analysed by hierarchical clustering and MDS analysis (Figure 2). In both analyses, accessions grouped together according to botanical variety indicating that allelic variation at these markers reflected well-established botanical varieties. Interestingly, var. *bituminosa *accessions were sub-divided into two clear groups according to geographical origin (Canary Islands and Mediterranean region). Cluster analysis revealed that some accessions were genetically so similar that they could not be distinguished using allele information at 21 SSR loci (Figure 2a). Heterozygosity of the original populations ranged from 0-62% (mean = 16.4%; Table 2). An analysis of molecular variance (AMOVA) confirmed that there was significant differentiation between populations accounting for 35% of the total allelic variance observed (Table 3).

**Table 3:** Analysis of molecular variance (AMOVA) in single plants sampled from 26 original populations of *Bituminaria bituminosa*

Source	df	SS	MS	**Est. Var**.	%
**Between Pops**	2	180.425	90.213	8.549*	35%

**Within Pops**	23	373.306	16.231	16.231*	65%

**Total**	25	553.731		24.780	100%

*Significant at *P *< 0.001

Three populations were defined as var. *albomarginata *(n = 9), var. *bituminosa *from the Canary Islands (n = 9) and var. *bituminosa *from the Mediterranean region (n = 8). Botanical variety *crassiuscula *was not included because there was only one accession used in this study.

### Using markers to characterise *B. bituminosa *breeding lines

**Figure 3:**
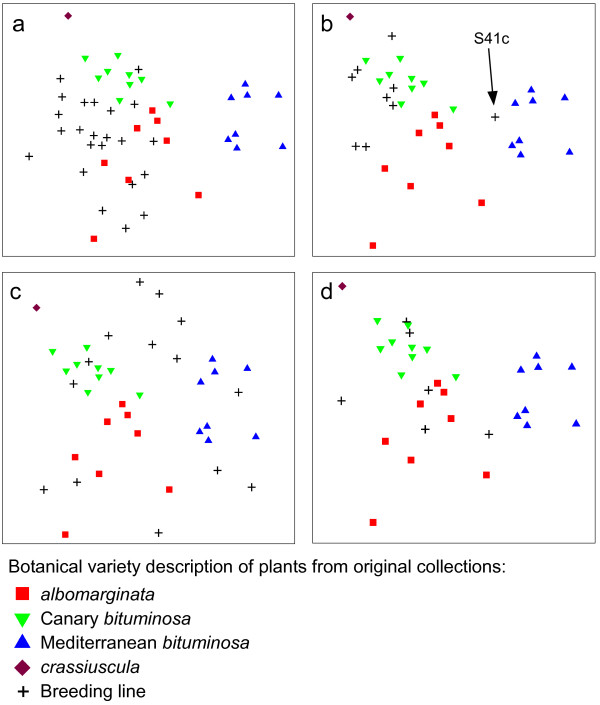
**Multidimensional scaling (MDS) plots of 79 *Bituminaria bituminosa *accessions from three botanical varieties from the Canary Islands and the Mediterranean region**. Each MDS plot was generated from pairwise Euclidean distances of all 79 accessions (2D stress = 0.23) with each plot showing the position of additional breeding lines (denoted by "+" signs) from botanical varieties: a) var. *albomarginata*; b) var. *bituminosa *(Canary Islands); c) var. *bituminosa *(Mediterranean region); and d) var. *crassiuscula*.

Having established that the usefulness of the new SSR markers, genotype information from an additional 52 *B. bituminosa *breeding lines was subjected to pairwise distance analysis, along with plants from the 27 original populations (Additional file 5). This distance matrix was analysed by MDS and hierarchical clustering techniques (Figure 3 and Additional file 6). Adding these lines to the analyses resulted in an increased complexity of inter-relationships as evidenced by an increase in the two-dimensional MDS stress from 0.16 (27 original populations) to 0.23 (all 79 accessions). However, a similar pattern of groupings among the original populations was observed (compare Figure 2b and Figure 3).

Figure 3a-3d shows the placement of breeding lines of three botanical varieties from the Canary Islands and Mediterranean region relative to the original populations. In general, breeding lines described as var. *albomarginata *and var. *bituminosa *(from the Canary Islands) were placed within or near their respective clusters of original populations with one notable exception: breeding line S41c (Figure 3b) was described as Canary Island *bituminosa *but had much greater affinity to Mediterranean *bituminosa *according to SSR marker genotyping. Breeding lines described as Mediterranean *bituminosa *and var. *crassiuscula *had very wide distribution in the MDS plots (Figure 3c and 3d). The average heterozygosity of breeding lines was substantially higher (32.0%) than for original populations (16.4%), and had a greater range of values (0-95%; Table 2).

## Discussion

New high-throughput DNA sequencing technologies offer many research opportunities for both model and crop species alike [15]. Arguably, minor grain and pasture crop species (also known as "orphan" crop species [19]) stand to gain the most since they are starting from a very low baseline of genomic resources. This paper describes a method for developing codominant and polymorphic genic SSR markers from a modest sampling of the leaf transcriptome derived from a single plant of *B. bituminosa *by Roche 454 pyrosequencing. This method was highly effective with 75 out of 87 (86.2%) automatically designed primer pairs successfully amplifying PCR products (Additional file 3). Of the 21 markers selected for high- resolution fragment analysis in plants from 79 *B. bituminosa *accessions, 20 gave consistently strong amplification products and were highly polymorphic (6.19 alleles per marker locus). The methods presented in this study could be used by researchers for other genomic orphan species for rapid development of high-quality codominant markers, although the extent of marker polymorphism will vary between species and between populations within species.

The usefulness of the SSR markers was demonstrated by a clear delineation of groups according to botanical variety and geographical location in plants sampled from 27 original populations. These markers advanced our understanding of genetic diversity in *B. bituminosa *in that we could clearly distinguish var. *bituminosa *types according to their geographical origin (Canary Islands and the Mediterranean region; Figure 2). This differentiation was of similar magnitude to that which distinguished botanical varieties and extends the observation by Juan et al. [4] who found that Mediterranean var. *bituminosa *formed its own grouping while all botanical varieties from the Canary Islands formed another group. Therefore, there is a *prima facie *case for dividing var. *bituminosa *into two botanical varieties according to geographic origin (Canary Island or Mediterranean region), each distinct from var. *albomarginata *and var. *crassiuscula*. There are some differences in trait characteristics that support this division. For example, Mediterranean var. *bituminosa *are usually biennial whereas Canary Island var. *bituminosa *are perennial.

These SSR markers provided some useful insights into *B. bituminosa *breeding lines. Historically, the *B. bituminosa *breeding programme has practiced uncontrolled open pollination. While most pollination in *B. bituminosa *is considered to be by self-pollination, a certain proportion of out-crossing does occur [21] but until now there has been little direct evidence to support this assumption. In this study, two lines of evidence were found to support the occurrence of out-crossing in the *B. bituminosa *breeding programme. First, the average level of heterozygosity increased approximately two-fold in breeding lines (32.0%) versus original populations (16.4%) (Table 2). Second, cross-pollination between *B. bituminosa *plants from different botanical varieties and geographical locations is the most likely explanation of the wider distribution of breeding lines compared to original populations in MDS plots (Figure 3). Interestingly, this increased distribution was most pronounced in Mediterranean var. *bituminosa*. This may in part be explained by breeder observations in Murcia (Spain) that populations of var. *bituminosa *presented a higher percentage of fruit set compared to populations of var. *albomarginata*, which was taken to be indirect evidence of higher cross-pollination frequencies in var. *bituminosa *[22]. The same observation was made for var. *crassiuscula*, though analysis of additional accessions is necessary to confirm this preliminary conclusion.

An advantage of using the transcriptome sequencing approach compared to more conventional genomic SSR approaches is that markers should be more transferable across species since they are based on gene sequences that are relatively well conserved in evolution compared to non-genic regions [23]. For example, these genic markers may prove useful in other Psoraleeae species that remain genomic orphans, such as *Cullen australasicum*, an Australian native perennial legume species that shows promise as a drought-tolerant pasture species [24].

In addition to generating useful SSR markers, this study provides a repertoire of many thousands of expressed gene sequences for potential follow-up experiments. Gene ontology analysis using Blast2GO was highly effective in identifying putative cellular location, biological process and molecular function in 3,419 out of 3,838 (89.1%) assembled mRNA sequences. Examples of follow-up experiments may include investigating genes associated with flowering time regulation (e.g. *CONSTANS*-like a [23]; isotig02726, Additional file 1) or furanocoumarin biosynthesis genes (e.g. cytochrome p450 monooxygenases [24,25]; e.g. isotig00288, Additional file 1). The species showing closest homology to *B. bituminosa *in 3,013 out of 3,419 (88.1%) mRNA sequences with significant hits was *G.max*, indicating that *G. max *will serve as a useful reference genome for future gene discovery and characterisation in *B. bituminosa*.

## Conclusions

This paper describes an efficient method for developing valuable SSR markers for *B. bituminosa*, a species that could previously be described as a genomic orphan. These markers gave new insight into genetic variation in *B. bituminosa*, providing evidence that a division of the botanical variety *bituminosa *may be appropriate. Evidence of cross pollination was found between botanical varieties in the *B. bituminosa *breeding programme. The expressed gene repertoire discovered in this experiment may be useful for follow-up experiments targeting biochemical pathways and/or important agronomic traits.

## Methods

### Selection of a *B. bituminosa *accession for transcriptome sequencing

In June 2008, one plant from each of 22 accessions was analyzed for psoralen and angelicin content (Ewald Sweeny, Chem Centre, Western Australia; unpublished data). The plant A13.1 (from accession A13) had the highest total content of furanocoumarins (highest in angelicin and third highest in psoralen) out of the 22 plants evaluated. This accession is from an original population collected from the Teno region of the Canary Islands. Plant A13.1 was cloned by propagating cuttings which were then kept at the Department of Agriculture and Food Western Australia (DAFWA) in a naturally lit glasshouse at a constant temperature of 25°C. One such clone was used for transcriptome sampling.

### mRNA extraction and cDNA preparation

Young leaves were harvested from A13.1 and immediately frozen in liquid nitrogen. Leaves were ground to a fine powder in liquid nitrogen using a mortar and pestle. Total RNA was extracted using RNeasy kit (Qiagen) and mRNA isolated using Oligotex mRNA kit (Qiagen). mRNA was purified using RNeasy Minelute kit (Qiagen) and quality checked at the Australian Genome Research Facility (AGRF; Brisbane, Australia) using an RNA6000 Pico chip (Agilent) run on a BioAnalyzer 2100 (Agilent). A cDNA library was constructed by AGRF following the standard Roche Diagnostics protocol ("cDNA Rapid Library Preparation Method Manual-GS FLX Titanium Series", October 2009 (Rev. Jan 2010)).

### Sampling the *B. bituminosa *transcriptome by Roche 454 sequencing

Sequencing of the cDNA library was carried out at AGRF using the GS-FLX System (Roche Diagnostics) with Titanium sequencing chemistry on one half of a two-region gasket PicoTitre Plate; for full details, consult the Roche Diagnostics "Sequencing Method Manual-GS FLX Titanium Series", October 2009 (Rev. Jan 2010). The GS De Novo Assembler software (Roche Diagnostics) was used to assemble the sequencing output into contigs, using default parameters.

### De novo assembly of *B. bituminosa *leaf transcriptome

The GS De Novo Assembler (version 2.3, Roche Diagnostics) software programs "newAssembly" (with "cdna" parameter) and "runProject" were used to align and assemble the sequencing output from Standard Flowgram Format into contigs and isotigs, using default parameters as described by the manufacturer.

### Functional Analysis

Gene ontology (GO) classification was conducted with the aid of Blast2GO software [26] using GenBank database version 173. Isotig and remaining singleton contigs > 100 bp were included in the analysis. Matches with significance values < 1e-6 were allocated to three GO categories (Biological Process, Molecular Function, and Cellular Component) in 1 to 11 levels of hierarchical structure. For ease of visualisation, results are presented at levels 2 or 3 (Figure 1).

### SSR detection and primer design

The raw GS-FLX sequencing output in FASTA format was submitted as input to the QDD program [27] for detection of SSR markers and primer design. The QDD program was run from the command line on a Linux system using the default parameters as described in the QDD user manual. For comparison, a manual search for repeat motifs among the isotig/contig sequences was performed and primers flanking a subset of repeats were designed with the aid of Geneious 5.3 (Biomatters Ltd).

### SSR marker amplification and fragment analysis

Genomic DNA was extracted from 50 *B. bituminosa *accessions provided by the Future Farm Industries Cooperative Research Centre (FFI CRC) at the Department of Agriculture and Food Western Australia (South Perth, Australia) and 29 *B. bituminosa *accessions provided by the Spanish Breeding programme at Instituto Murciano de Investigación y Desarrollo Agrario y Alimentario (Murcia, Spain) (Table 2), using Illustra Nucleon Phytopure Genomic DNA Extraction Kits (GE Healthcare). PCR reactions were carried out in a MasterCycler programmable thermal cycler (Eppendorf) in 20 uL volumes containing the following components: 2.5 ng/uL genomic DNA, 1x PCR buffer (comprising 50 mM KCl, 10 mM Tris HCl (pH 9.0) and 0.1% Triton-X), 2 mM MgCl_2_, 200 μM dNTPs, 0.04 U/uL Taq DNA polymerase and 0.2 μM each of forward and reverse primers. Amplification conditions consisted of denaturation at 94°C for 5 min followed by 35 cycles of denaturation at 94°C for 45 sec, primer annealing at 55°C for 45 sec and extension at 72°C for 90 sec, followed by a final extension step of 72°C for 7 min.

Initial screening of 96 SSR primer pairs for amplification efficiency was carried out using genomic DNA from plant A13.1 that had been used for transcriptome sequencing. Primer pairs that successfully amplified fragments in A13.1 (assessed using conventional TBE agarose electrophoresis) were then used to screen eight diverse *B. bituminosa *accessions (individual plants: A13.1, A27.2, A37.2, A42.3, A43.2, A48.2, A51.2 and S2b) for polymorphism. For those markers identified as polymorphic on 2% TBE agarose gels, fluorescently-labelled forward primers were synthesised and used to amplify fluorescently labelled amplicons in 79 lines. Fragment size analysis was performed relative to a Genescan LIZ500 internal size standard (Applied Biosystems) using an AB3730xl capillary DNA sequencer (Applied Biosystems) with the resulting electropherograms analysed using GeneMarker software (SoftGenetics) as described in detail by Nelson et al. [28]. Each marker allele was recorded as estimated base pair length. For genetic distance estimates, alleles were scored as present (1), absent (0) or unknown (999).

### Genetic diversity analyses

Pairwise Euclidean distances among 79 *B. bituminosa *accessions (Table 2) were calculated using NTSYSpc 2.21i (Applied Biostatistics Inc.). Pairwise distances were subjected to hierarchical cluster analysis using group averages and multidimensional scaling (MDS) using Kruskal fit scheme 1 with 100 restarts in Primer 6.1.6 software (Primer-E Ltd). Analysis of Molecular Variance (AMOVA) of variation within and among original populations (n = 26, excluding var. *crassiuscula *as there was only one accession represented) and allele frequencies (n = 27) were calculated using GenAlEx 6.4 [29]. Polymorphism information content (PIC) was calculated using the formula described by Pradhan et al. [30].

## Authors' contributions

MPN carried out all laboratory work, except for RNA and mRNA extractions for the sequenced library, which were performed by NT, MD, EC and DR collected and characterised *B. bituminosa *germplasm and selected plants for DNA and RNA sampling. KH selected and implemented bioinformatic analyses. MNN planned and supervised the laboratory work, with additional supervision provided by NT. MPN and MNN conducted genetic diversity analyses. All authors contributed to the final version of the manuscript.

## Supplementary Material

Additional file 1 **Gene ontology (GO) terms for 3,419 *Bituminaria bituminosa *mRNA sequences**. GO terms with associated descriptions generated using Blast2GO software. Click here for file

Additional file 2 **240 primer pairs targeting simple sequence repeat motifs**. Primer pairs designed using Primer3 based on Roche 454 sequences identified as containing simple sequence repeats using QDD software. Click here for file

Additional file 3 **Primer pairs selected for testing**. Details of 94 primer pairs targeting simple sequence repeat (SSR) motifs in transcribed genes of *Bituminaria bituminosa *leaves sampled by Roche 454 transcriptome sequencing. Click here for file

Additional file 4 **Allele frequencies in *Bituminaria bituminosa *plants**. A total of 118 simple sequence repeat (SSR) marker alleles were detected using 19 single-locus markers. Marker *Bbit*-SSR079 detected two loci and so was not included here. Polymorphic information content (PIC) and allele frequencies were calculated based on the whole population of 79 *B. bituminosa *plants. Allele frequencies are also presented for four original populations of botanical varieties *albomarginata, bituminosa *(from the Canary Islands and Mediterranean region) and *crassiuscula *that together comprised 27 plants. Click here for file

Additional file 5 **Euclidean distance matrix**. 79 *Bituminaria bituminosa *plants were genotyped at 21 simple sequence repeat loci. The resulting 130 alleles were used to calculate pairwise Euclidean distances. Click here for file

Additional file 6 **Cluster tree of 79 *Bituminaria bituminosa *plants**. Based on Euclidean distances estimated using 130 simple sequence repeat marker alleles. Symbols indicate botanical variety and type of line (original collection or breeding line). Click here for file
