# Supplementary material for: Next generation DNA sequencing technology delivers valuable genetic markers for the genomic orphan legume species, Bituminaria bituminosa
Source: BMC Genet. 2011 Dec 15;12:104. doi: 10.1186/1471-2156-12-104 (PMC3265443; doi:10.1186/1471-2156-12-104)

Additional file 6 - Cluster tree of 79 *Bituminaria bituminosa* plants. Based on Euclidean distances estimated using 130 simple sequence repeat marker alleles. Symbols indicate botanical variety and type of line (original collection or breeding line).

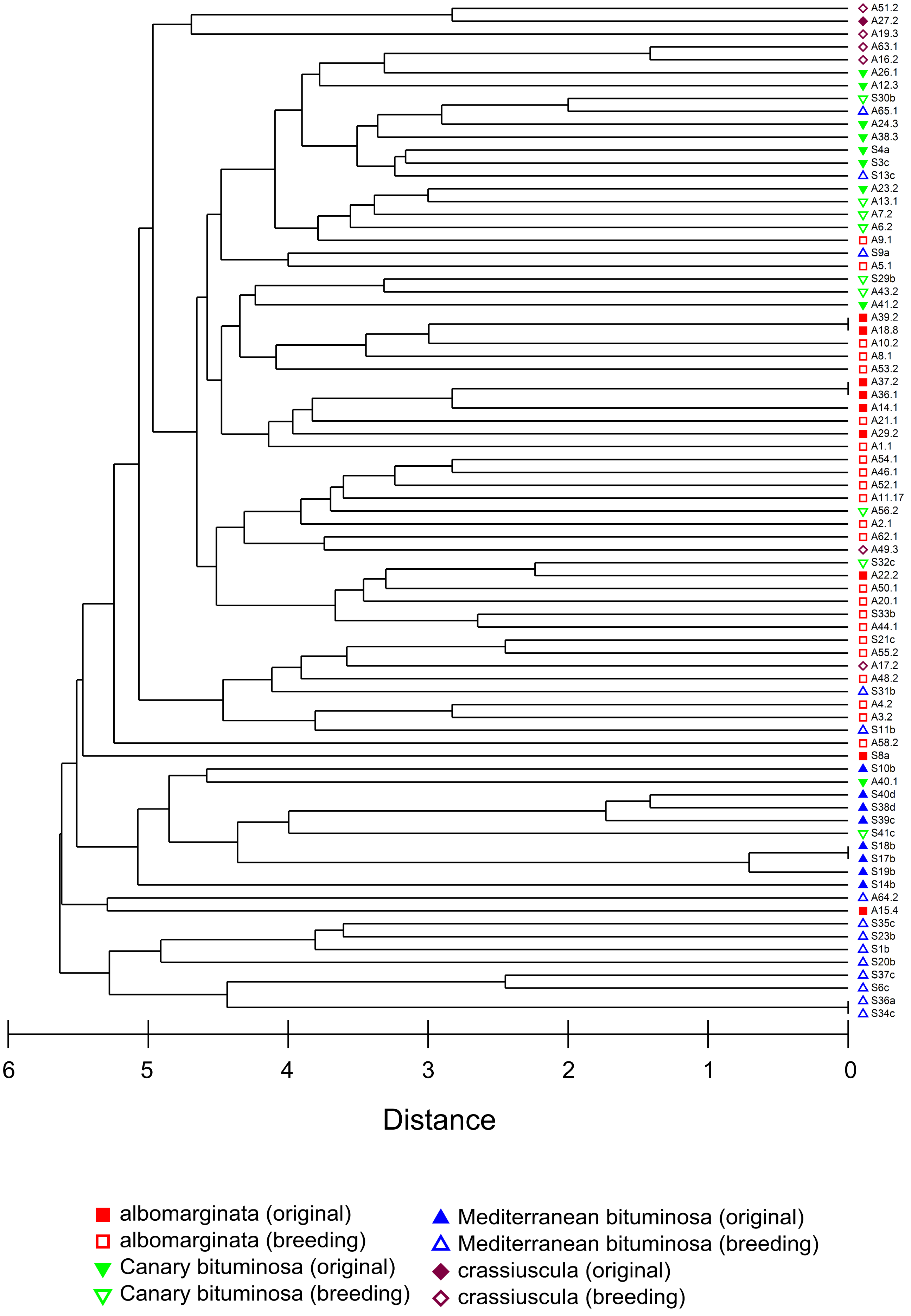

Supplement: Additional file 6 — Cluster tree of 79 Bituminaria bituminosa plants. Based on Euclidean distances estimated using 130 simple sequence repeat marker alleles. Symbols indicate botanical variety and type of line (original collection or breeding line). [file 1471-2156-12-104-S6.PDF]
